# Moral distress and its contribution to the development of burnout syndrome among critical care providers

**DOI:** 10.1186/s13613-017-0293-2

**Published:** 2017-06-21

**Authors:** Renata Rego Lins Fumis, Gustavo Adolpho Junqueira Amarante, Andréia de Fátima Nascimento, José Mauro Vieira Junior

**Affiliations:** 1 Intensive Care Unit, Hospital Sírio-Libanês, São Paulo, SP Brazil; 2 Department of Public Health, Faculdade de Ciências Médicas, Santa Casa de São Paulo, São Paulo, SP Brazil

## Abstract

**Background:**

Burnout appears to be common among critical care providers. It is characterized by three components: emotional exhaustion, depersonalization and personal accomplishment. Moral distress is the inability of a moral agent to act according to his or her core values and perceived obligations due to internal and external constraints. We aimed to estimate the correlation between moral distress and burnout among all intensive care unit (ICU) and the step-down unit (SDU) providers (physicians, nurses, nurse technicians and respiratory therapists).

**Methods:**

A survey was conducted from August to September 2015. For data collection, a self-administered questionnaire for each critical care provider was used including basic demographic data, the Maslach Burnout Inventory (MBI) and the Moral Distress Scale-Revised (MDS-R). Correlation analysis between MBI domains and moral distress score and regression analysis to assess independent variables associated with burnout were performed.

**Results:**

A total of 283 out of 389 (72.7%) critical care providers agreed to participate. The same team of physicians attended both ICU and SDU, and severe burnout was identified in 18.2% of them. Considering all others critical care providers of both units, we identified that overall 23.1% (95% CI 18.0–28.8%) presented severe burnout, and it did not differ between professional categories. The mean MDS-R rate for all ICU and SDU respondents was 111.5 and 104.5, respectively, *p* = 0.446. Many questions from MDS-R questionnaire were significantly associated with burnout, and those respondents with high MDS-R score (>100 points) were more likely to suffer from burnout (28.9 vs 14.4%, *p* = 0.010). After regression analysis, moral distress was independently associated with burnout (OR 2.4, CI 1.19–4.82, *p* = 0.014).

**Conclusions:**

Moral distress, resulting from therapeutic obstinacy and the provision of futile care, is an important issue among critical care providers’ team, and it was significantly associated with severe burnout.

**Electronic supplementary material:**

The online version of this article (doi:10.1186/s13613-017-0293-2) contains supplementary material, which is available to authorized users.

## Background

Burnout has been described by Maslach and coworkers as a psychological syndrome arising in response to chronic interpersonal stressors on the job, A condition in which professionals “lose all concern, all emotional feeling for the people they work with, and come to treat them in a detached or even dehumanized way” [1].

Burnout is widely described in its tridimensionality: (1) emotional exhaustion, (2) depersonalization and (3) personal accomplishment [2–4]. High levels of burnout have been found among healthcare professionals in ICUs, with prevalence rates ranging from 0 to 70% [5].

Although there are significant professional repercussions including decreased patient satisfaction, increased medical errors and disagreements, and the personal consequences of substance abuse and depression [3–5], burnout may be due to the burden of impotence related to hierarchical power structures, ineffective or obstructive policies, dysfunctional communication patterns, lack of resources and other issues beyond the providers control [6].

Moral distress is defined as the inability of a moral agent to act according to his or her core values and perceived obligations due to internal and external constraints [7]. Although it was originally conceptualized to address ethical issues in nursing [8–12], all healthcare professionals might face morally relevant questions pertaining to the “rightness” or “wrongness” of decisions, treatments or procedures, while feeling powerless to change situations they perceive as morally wrong [7].

Furthermore, moral distress and burnout are close phenomena and one may suspect that determinants are similar, such as the association of burnout with experiencing a patient’s death and with an ethical decision making [8, 12, 13]. As such, specialists suggest that the most damaging consequence of moral distress is in fact burnout [6, 14]. However, since there are no studies speculating about this correlation among all critical care providers, from different units, the aims of this study are:

1. To estimate both burnout syndrome and moral distress prevalence and severity among all ICU providers (physicians, nurses, nurse technicians and respiratory therapists) and compare them with the step-down unit providers.
2. To estimate the correlation between moral distress and burnout.

## Methods

### Setting

This survey was conducted in the tertiary, private, teaching, 470-bed Hospital Sírio-Libanês, in São Paulo, Brazil. The institutional review board (IRB), called “Comitê de Ética em Pesquisa da Sociedade Beneficiente de Senhoras do Hospital Sírio-Libanês,” reviewed and approved this study (HSL protocol number HSL 2015-65, 07/14/2015).

ICU providers (physicians, nurses, nurse technicians and respiratory therapists) and also the SDU providers working for more than 6 months in the unit were invited to participate and all signed a written informed consent. The intensive care unit (ICU) comprises 22 private rooms for general medical–surgical care and eight private rooms for neurological care. The professional-to-bed ratios in the ICU is: nurse 1:4; nurse technicians 1:2; physician 1:6 (day shift) and 1:10 (night shift). The ICU has a 24-h visitation policy (day or night free entry, with possibility to change the visitor at any time, and option to sleep with the patient in an individual box).

The step-down unit (SDU) provides an intermediate level of care between the intensive care units (ICUs) and the general medical–surgical wards. In our hospital, it consists in a 31-bed unit with monitoring and presence of a physician belonging to the same team of intensivists (ratio of physician: patient 1:8 mornings and 1:15 during in the rest of the day; nurse 1:6 and nurse technicians 1:3).

### Interviews

For data collection, a self-administered questionnaire for each critical care provider was used including basic demographic data and two more instruments: (1) the Maslach Burnout Inventory (MBI) to assess burnout and (2) Moral Distress Scale-Revised (MDS-R) to investigate moral distress. All workers who agreed to participate in this study returned the questionnaire in a sealed envelope.

The MBI is a 22-item questionnaire that has shown to be reproducible and valid [15]. The MBI evaluates three domains of burnout: The emotional exhaustion subscale (nine items) assesses feelings of being emotionally overextended and exhausted by work; the depersonalization subscale (five items) measures an unfeeling and impersonal response toward recipients of one’s service, care or treatment; the personal accomplishment subscale (eight items) assesses feelings of competence and successful achievement in one’s work with people.

Cutoff scores were defined for each dimension, and we adopted the following internationally established definition of burnout, according to the MBI manual: high levels of emotional exhaustion (score ≥ 27 points) and depersonalization (score ≥ 10 points) combined with low personal accomplishment (score ≤ 33 points) [16].

We also used the Standard Hamric Moral Distress Scale-Revised (MDS-R) [17]. We previously obtained the author’s authorization for use of the MDS-R. Prior to use, the scale was translated into Portuguese using the forward–backward method and then matched. All questions were translated into Portuguese and validated before being applied [18]. The instrument was translated by two bilingual experts, and then, a back translation was made by other two translators who had not seen the original scale, in order to verify the equivalence of terms between the two versions. After translation and adaptation, the authors ensured that the new version included the evaluation properties required for application.

The Moral Distress Scale-Revised (MDS-R) is a questionnaire that measures moral distress in specific situations: Respondents are asked to indicate both the frequency (“*F*”) and the level of disturbance (intensity = “*I*”) when the situation arises.

The items of MDS-R were categorized according to Likert scale from zero (never) to 4 (often) to assess the frequency of moral distress and from zero (none) to 4 (largely) to assess the intensity of moral distress, which can range from 0 to 16, where the items that are less distressing have low “*f*” × “*i*” scores versus more distressing items, which have higher “*f*” × “*i*” scores. First, the product of frequency and intensity is obtained: [“*F*” × “I”] means *F* (frequency) multiplied by *I* (intensity) and the final value = [(frequencies score) × (intensity score)] is obtained, allowing to identify individual items or situations that are distressing. Then, the Likert scale data can be computed into a composite score or actual moral distress using a two-part procedure. The final composite is obtained by summing each item’s [“*F*” × “*I*”] score, resulting in a range of 0–336, where less actual distress has low composite score and more actual moral distress yields higher composite score [17].

### Statistical analysis

Descriptive analysis was performed using mean and standard deviation (or median and interquartile range for nonparametric variables) for quantitative variables and frequencies for categorical variables. We compared staff characteristics according to workplace using the Chi-square test, Student’s *t* test and Wilcoxon rank-sum test, when appropriated. We calculate prevalence and its respective 95% confidence interval (95% CI) for severe burnout among critical care providers. Prevalence ratios (PR), their respective 95% CI and Chi-square test were used to compare severe burnout prevalence according to subjects’ characteristics.

Moral distress scores among professional categories were compared using one-way ANOVA; to compare these scores between workplaces, we performed *t* test. We used Pearson’s correlation coefficient to examine the correlation between MBI domains of burnout and MDS-R overall score. Comparisons between moral distress individual questions answers according to the presence or absence of severe burnout were made using Wilcoxon rank-sum test. We performed a logistic model step by step and analyses by Hosmer–Lemeshow test. Variables with *p* value <0.20 in univariate analysis were tested in the final model. A *p* value < 0.05 was considered significant. Statistical analysis was performed using the Stata^®^ 13.1 software (Stata Corp LP, College Station, TX, USA).

## Results

### Participants

A total of 283 of 389 (72.7%) critical care providers agreed to participate, and the survey was conducted in August and September 2015. Out of this total, 134 were from ICU and 116 from a step-down unit. Regarding physicians, the same team of intensivists attended both ICU and step-down unit and they were treated as an individual group. Thirty-three of them (67.3%) participated, representing 11.7% of the total respondents. Nurses 35/134 (26.1%) were from ICU and 28/116 (24.1) from SDU, nurse technicians 68/134 (50.7%) from ICU and 60/116 (51.75%) from SDU and respiratory therapists 30/134 (22.4%) from ICU and 27/116 (23.3%) from SDU.

### Physicians characteristics

Regarding physicians, 66.6% were male; mean age was 38.71 ± 6.65 years; all of them have a personal income of more than $2.350; none spent more than 1 h on the way to work; 66.6% were working night shift and 63.6% had more than one job. They had 8.33 ± 7.06 years of work in the unit. We observed that the majority (84.8%) had more than 10 h per week of leisure; 75.7% had sexual activities more than once a week; 63.6% did regular physical activity; 48.4% had a regular hobby; and 12.1% were doing psychotherapy at the time of interview. Regarding religion, 51.5% were Catholics followed by atheists (21.2%) and Spiritualist religion (15.1%).

Table 1 describes the characteristics of the other healthcare providers of both ICU and SDU.

**Table 1:** Characteristics of healthcare providers according to their workplace

Characteristics	ICU^a^ n (%)	Step-Down Unit *n* (%)	*p*
Female gender	101/132 (76.5)	77/113 (68.1)	0.143*
Age—mean (SD^b^)	35.5 (7.1)	34.4 (6.6)	0.224**
Marital status—married	76/130 (58.5)	71/115 (61.7)	0.601*
Night shift	58/126 (46.0)	56/115 (48.7)	0.679*
Time spent on the path to arrive at work >60 min	55/133 (41.4)	56/115 (48.7)	0.246*
Catholic religion	67/133 (50.4)	49/113 (43.4)	0.272*
Personal income >R$5000	30/131 (22.9)	24/113 (21.2)	0.755*
Family support	78/130 (60.0)	65/110 (59.1)	0.886*
Time working at the institution (years)—median (p25–p75)^c^	6.0 (3.0–12.0)	4.0 (2.0–9.0)	0.001***
Has a non-care activities in the institution	16/130 (12.3)	9/109 (8.3)	0.308*
Psychiatric treatment	9/133 (6.8)	7/114 (6.1)	0.842*
Psychotherapy	12/133 (9.0)	1/115 (0.9)	0.004*
Works out of hospital	42/133 (32.6)	36/114 (31.6)	>0.999*
Absenteeism (last month)	33/133 (24.8)	29/115 (25.2)	0.941*
Tobacco use	16/132 (12.1)	7/115 (6.1)	0.104*
Alcohol use ≥1 drink/week	27/133 (20.3)	27/115 (23.5)	0.545*
Leisure ≥5 h/week	49/131 (37.4)	35/114 (30.7)	0.270*
Regular physical activity	45/130 (34.6)	50/109 (45.9)	0.077*
Has regular hobbies	45/108 (41.7)	46/95 (48.4)	0.334*
Sexual activity ≥1 time/week	86/132 (65.1)	78/113 (69.0)	0.520*

* Chi-square test

** Student’s *t* test

*** Wilkoxon rank-sum test

^a^Intensive care unit

^b^Standard deviation

^c^25th–75th percentiles

We observed that the categories of respiratory therapists (64%) and physicians (64%) have more than one job, significantly higher than the nurses (14%) and nurse technicians (24%), (*p* < 0.0001).

We did not find a difference in burnout prevalence in the healthcare providers who worked night shifts, compared with others 24.3% (*n* = 33) versus 20.4% (*n* = 28), *p* = 0.448. Regarding the number of working hours, due to local rules, within each category, professionals have similar workloads.

### Prevalence of severe burnout among professional categories

Three respondents did not complete the MBI. Overall, the prevalence of severe burnout was 22.5% (95% CI 17.7–27.8%). Severe burnout was identified in 18.2% of physicians. Considering all other critical care providers (ICU + SDU), we identified that 23.1% (95% CI 18.0–28.8%) presented severe burnout. There was no statistical difference between the units: From ICU respondents, 29/131 (22.1%) had severe burnout and 27/114 (23.7%) from SDU (*p* = 0.774).

Additional file 1: Figure S1 shows the prevalence of severe burnout in all respondents, according to the professional category. The differences found in the burnout rate in the SDU were nurse = 39.3%, nurse technicians = 15.2% and respiratory therapists = 25.9% with *p* = 0.046 and in the ICU were nurse = 28.6%, nurse technicians = 20.9% and respiratory therapists = 16.6% with *p* = 0.490. We did not find any differences in prevalence of severe burnout between workplace when comparing nurses (*p* = 0.370), nurse technicians (*p* = 0.391) or respiratory therapists (*p* = 0.392). Although the frequency of burnout in ICU + SDU (Additional file 1: Figure S1) was higher in nurses (33.8%), no significant difference was found between the different categories (*p* = 0.093).

The score for emotional exhaustion ranged from 9 to 45 (mean = 26.4; SD = 8,1); for depersonalization, the score ranged from 5 to 22 (mean = 9.8; SD = 3.9) and for personal accomplishment from 21 to 40 (mean = 33.1; SD = 4.5). Whereas depersonalization was similar among all professionals, we observed high rates of emotional exhaustion in MBI subscale much more often among nurses (60%) compared with respiratory therapists (50.8%), nurse technicians (43.6%) and physicians (27.3%), *p* = 0.015. We also more frequently identified in the nurses category lower level of personal accomplishment (61.5%) compared with others professionals (*p* = 0.018) (Additional file 2: Figure S2).

### Moral distress

Exploratory factor analyses were performed by Kaiser–Meyer–Olkin measure of sampling adequacy (0.852) and Bartlett’s test of sphericity (*p* < 0.0001). Also, the Cronbach’s alpha was calculated and presented a good performance value 0.897.

A total of 68 questionnaires were incomplete and excluded from analysis. The category with more incomplete questionnaire was nurse technicians (33.6%) followed by nurses (21.5%), respiratory therapists (12.3%) and physicians (12.1%), *p* = 0.004.

All respondents (*n* = 215) had a response rate of 55%: 99 were from ICU; 87 were from SDU; and 29 physicians reported an overall high level of moral distress (mean score 107.6; SD 59.2; range 6–292); 104 (48.4%) respondents had moral distress score >100 (95% CI 41.5–55.3%). The mean MDS score for all ICU respondents including both domains (frequency and intensity) was 111.5 (SD 57.6; range 27–292) and for all SDU respondents was 104.5 (SD 66.0; range 6–264) (*p* = 0.446). We observed similar mean MDF score distribution among professionals (105.6 ± 42.0 for physicians, 107.7 ± 60.4 for nurses, 109.9 ± 63.2 for nurse technicians and 104.9 ± 60.9 for respiratory therapists, *p* = 0.967). Regarding moral distress intensity, we found a higher rate in ICU compared with the step-down unit (43.8 ± 16.9 vs 37.2 ± 17.4, *p* = 0.010) (Table 2).

**Table 2:** Moral Distress Scale-Revised scores according to professional category and workplace

	Physicians (*n* = 29) Mean (SD^a^)	Nurses (*n* = 51) Mean (SD)	Nurse Technicians (*n* = 85) Mean (SD)	Respiratory therapists (*n* = 50) Mean (SD)	p*	ICU^b^ (*n* = 99) Mean (SD)	Step-down unit (*n* = 87) Mean (SD)	p**
Frequency	39.4 (9.8)	40.0 (13.7)	39.2 (14.3)	38.4 (13.8)	0.946	39.2 (13.3)	39.2 (14.7)	0.992
Intensity	47.8 (12.4)	40.7 (16.3)	40.5 (17.8)	41.1 (18.0)	0.219	43.8 (16.9)	37.2 (17.4)	0.010
Total	105.6 (42.0)	107.7 (60.4)	109.9 (63.2)	104.9 (60.9)	0.967	111.5 (57.6)	104.5 (66.0)	0.446

* One-way ANOVA

** Student’s *t* test

^a^Standard deviation

^b^Intensive care unit

### The correlation between burnout and moral distress

**Fig. 1:**
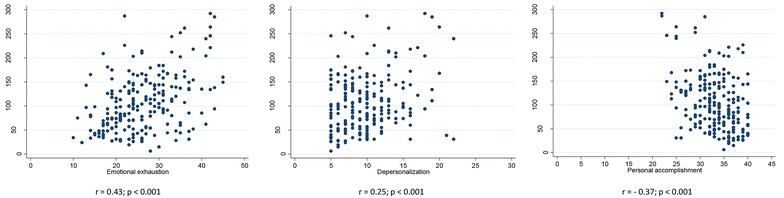
Correlations between the Maslach Burnout Inventory domains and MDS-R scores (*n* = 215)

Several questions from the MDS-R questionnaire were significantly associated with severe burnout, as listed in Table 3. MDS-R score was moderately correlated with emotional exhaustion (*r* = 0.43; *p* < 0.001) (Fig. 1). There were an inverse weak correlation between MDS-R score and personal accomplishment (*r* = −0.37; *p* < 0.001) and also a very weak correlation between MDS-R score and depersonalization (*r* = 0.25; *p* < 0.001).

**Table 3:** Questions of Moral Distress Scale-Revised associated with burnout applied in the all professionals (ICU + SDU)

Moral distress questions	No burnout Median [IR^a^]	Severe burnout Median [IR]	p*
Provide less than optimal care due to pressures from administrators or insurers to reduce costs	0 [0–2]	1 [0–6]	0.009
Initiate extensive lifesaving actions when I think they only prolong death	6 [2–9]	7 [4–16]	0.005
Follow the family’s request not to discuss death with a dying patient who asks about dying	4 [1–9]	8 [4–12]	0.005
Continue to participate in care for a hopelessly ill person who is being sustained on a ventilator, when no one will make a decision to withdraw support	8 [4–12]	12 [7–16]	0.006
Avoid taking action when I learn that a physician or nurse colleague has made a medical error and does not report it	1 [0–4]	2 [1–4]	0.017
Assist a physician who, in my opinion, is providing incompetent care	4 [1–8]	4 [2–9]	0.013
Be required to care for patients I do not feel qualified to care for	0 [0–2]	1 [0–4]	0.041
Provide care that does not relieve the patient’s suffering because the physician fears that increasing the dose of pain medication will cause death	2 [0–8]	6 [2–12]	<0.001
Follow the family’s wishes for the patient’s care when I do not agree with them, but do so because of fears of a lawsuit	2 [0–6]	7 [1.5–12]	<0.001
Work with nurses or other healthcare providers who are not as competent as the patient care requires	4 [2–9]	6 [3–12]	0.035
Witness diminished patient care quality due to poor team communication	4 [2–9]	9 [3–16]	0.004

* Wilcoxon rank-sum test

^a^Interquartile range

According to ROC curve analysis, we observed that the MDS-R score was equal or higher than 100 points had a sensitivity of 68% and specificity of 57%, with a relative prevalence of 2.0, 95% CI 1.16–3.44, *p* = 0.010. Therefore, there is a fairly good association between the two instruments.

The variable moral distress is an independent predictor for severe burnout with adjusted odds ratio 2.8 (1.5–5.2) with good performance for validation model adjusted Hosmer–Lemeshow test (*p* = 0.998). No other variables showed association with severe burnout at univariate analysis (Additional file 3: Table S1). See the MDS-R Scale in Additional file 4.

## Discussion

The central point of this study was that we assessed all critical care providers (physicians, nurses, nurse technicians and respiratory therapists) from both ICU and SDU to evaluate the prevalence of burnout syndrome and moral distress, and their association. The most important finding in our study was that moral distress was significantly associated with severe burnout. This study contributes to the growing body of evidence that burnout and moral distress are actually close phenomena.

In contrast to previous studies, in which the prevalence of severe burnout is of nearly 50% among critical care physicians [3] and one-third of critical care nurses [4], we found that physicians were the category that presented less burnout (18%) while nurses followed by respiratory therapists were the categories with the highest prevalence of burnout. One possible explanation for the low burnout prevalence among physicians compared with nurses may be due to the fact that physicians share the burden of decision making and care of patients in group and with the attending physicians. Another possible explanation is that the definition of burnout differs across studies. Although some studies have used the Poncet definition (MBI score > −9) [4], we chose to use a score according to the MBI manual high levels of emotional exhaustion and depersonalization combined with low scores on Personal Accomplishment) [16], which has also been done by other investigators [14, 19, 20]. In addition, the three-dimensional structure of the MBI is likely to provide more precise answers, leading to focused interventions.

Merlani et al., in a multicenter Swiss National study, found that nurse assistants (41%), followed by physicians (31%) and nurses (28%), had a high degree of burnout. Interestingly, the rate of a high degree of burnout among the ICU center ranged from 5 to 62%, with a mean of 28%, similar to our results (22.1% with severe burnout in the p-ICU) [21].

Moreover, the definition of burnout differs across studies. Although some studies have used the Poncet definition (MBI score > −9) [4, 21], we chose to use a score according to the MBI manual (high levels of emotional exhaustion and depersonalization combined with low scores on Personal Accomplishment) [16], which was also done by other investigators [14, 19, 20]. In addition, the three-dimensional structure of the MBI is likely to provide more precise answers, leading to focused interventions. This is the most common, widely described and internationally validated instrument used to assess all three dimensions of burnout [13].

In our study, whereas depersonalization was similar among all professionals, in accordance with previous studies, our study revealed that nurses presented high rates of emotional exhaustion and lower levels of personal accomplishment compared with other professionals [22]. Various studies have demonstrated that nursing is stressful and that the incidence of burnout in this profession is elevated due to their high demands, low resources, interprofessional conflicts, among other problems [14, 23, 24].

The present data differ from those of previous studies [2–4], in which determinants of severe burnout syndrome were associated with demographic characteristics, such as a high number of working hours and night shifts. Furthermore, we thoroughly sought to investigate many variables potentially involved in burnout, for instance, commuting distance, income, leisure time, family support and sexual activities, and we still were not able to find any demographic factor associated with severe burnout.

In addition, although we have interviewed the personnel of two units having distinct characteristics, we did not find any demographic significant difference according to the workplace. This similarity might be explained partially because both units care for severe, demanding patients and families, and the providers take care of a comparable number of critically chronic ill patients, who need a prolonged support. Further, both units have a rather open format of structural organization, which means that intensivists have to share with the attending physician important decisions regarding admission, discharge and withhold/withdraw of support.

Our findings demonstrate a significant, positive relationship between moral distress and burnout. Interestingly, we could observe that all providers scored similarly regarding moral distress, notwithstanding the highest degree of burnout in nurses. According to the literature, this association appears to be related to the performance of the nurse’s role as advocate of the patient. The nurse is usually identified as an essential source of many dilemmas, such as conflicts between legal and ethical obligations, perceived powerlessness, power distance, workload, perception of inadequate medical treatment and failed communication by the medical team [14, 25].

According to the regression analysis, moral distress was an independent predictor for severe burnout. It is possible that moral distress resulting from the moral atmosphere could lead to internal constraints such as self-doubt, lack of self-assurance, fear, anxiety and other situations that predispose to burnout.

It is noteworthy that items of moral distress such as situations in patients’ suffering, prolonging life, poor team communication, medical error and feeling of incompetence were associated with burnout. Almost 40 % of MDS-R questions that were associated with burnout were related to end-of-life decision making (see Table 3). Accordingly, it has been suggested that moral distress resulting from therapeutic obstinacy, that is, the implementation of potentially non-beneficial treatments, seems to have an important influence on the development of burnout [26–28].

Poncet et al. [4] identified severe burnout in one-third of ICU nursing staff and one of the domains associated with severe burnout were end-of-life-related factors, such as caring for a dying patient. It is important to point out that our results agree with those of other studies, showing that terminal care can be one of the most important drivers of burnout and moral distress [5, 8, 29–32].

Our study has some limitations. The most important is that conflicts were not assessed, since according to previous studies, higher burnout levels were significantly associated with the occurrence of conflicts [3, 13]. Furthermore, conflicts result mainly from disagreements about treatment, ethical decision making and end-of-life care and therefore were identified in literature as a major burnout risk factor [3, 13].

Second, the study was conducted in a single center, with unique characteristics. Third, a meaningful numbers of invitees who did not respond the MDS-R could have changed the results, had they answered. There is a chance that recall bias might have occurred, as those with burnout syndrome are more prone to recall events associated with moral distress. Fourth, selection bias might have played a role, as those who agreed to participate (and thus responded) did it exactly because they might have been suffering from burnout syndrome. Yet, the other way around might possibly be true: Those with burnout did not accept to participate. Finally, given the cross-sectional study design, we can only infer the causal relationship between burnout and moral distress syndromes.

## Conclusion

Severe burnout syndrome is present in all critical care providers . A positive relationship was found between burnout and moral distress, and after regression analysis, moral distress was independently associated with burnout. We observed that all professional categories had a high and similar score of moral distress.

## Additional files

**Additional file 1: Figure S1.** Prevalence of severe burnout among professional categories according to workplace.

**Additional file 2: Figure S2.** Prevalence of severe burnout components among professional categories.

**Additional file 3: Table S1.** Respondents characteristics and their associations with severe burnout in all critical care providers. (n = 280)^#^

**Additional file 4:** Moral Distress Scale-Revised (MDS-R).

## Abbreviations

ICU: intensive care unit
SDU: step-down unit
MBI: Maslach Burnout Inventory
MDS-R: Moral Distress Scale-Revised
ANOVA: analysis of variance
ROC curve: receiver operating characteristics curve
