# Supplementary material for: Moral distress and its contribution to the development of burnout syndrome among critical care providers
Source: Ann Intensive Care. 2017 Jun 21;7:71. doi: 10.1186/s13613-017-0293-2 (PMC5479870; doi:10.1186/s13613-017-0293-2)
Supplement: Supplementary file 3 — Additional file 3: Table S1. Respondents characteristics and their associations with severe burnout in all critical care providers. (n = 280)# [file 13613_2017_293_MOESM3_ESM.docx]

**Table S1 –Respondents characteristics and their associations with severe burnout in all critical care providers. (n = 280)^#^.**

| **Characteristics** | **No Severe Burnout**  **(n = 217)**  **n (%)** | **Severe Burnout**  **(n = 63)**  **n (%)** | **PR¹ (95%CI²)** | **p*** |
| --- | --- | --- | --- | --- |
| Gender  Male  Female | 69 (78.4)  146 (76.8) | 19 (21.6)  44 (23.2) | 1  1.07 (0.67-1.72) | 0.772 |
| Age (years)  ≤ 29  30-39  40-49  >50 | 39 (78)  113 (75.3)  51 (82.3)  8 (72.7) | 11(22)  37 (24.7)  11 (17.7)  3 (27.3) | 1  1.12 (0.62-2.02)  0.80 (0.38-1.70)  1.24(0.41-3.71) | 0.719 |
| Marital status  Married  Single | 88 (81.5)  128 (75.3) | 20 (18.5)  42 (24.7) | 1  1.34 (0.83-2.14) | 0.227 |
| Personal income > $1.500  No  Yes | 147 (77.8)  67 (76.1) | 42 (22.2)  21 (23.9) | 1  1.07 (0.68-1.70) | 0.762 |
| Religion  Catholic  Others | 109 (75.7)  106 (79.1) | 35 (24.3)  28 (20.9) | 1  0.86 (0.55-1.83) | 0.497 |
| Professional category  Physicians  Nurses  NT  RT | 27 (81.8)  43 (66.1)  103 (81.7)  45 (78.9) | 6 (18.2)  22 (33.9)  23 (18.3)  12 (21.1) | 1  1.86 (0.84-4.14)  1.01 (0.44-2.26)  1.16 (0.48-2.79) | 0.088 |
| Workplace  Step Down Unit  ICU³ | 87 (76.3)  103 (78.0) | 27 (23.7)  29 (22.0) | 1  0.93 (0.58–1.47) | 0.749 |
| Time spent on the path to arrive at work  <60 minutes  ≥60 minutes | 132 (76.7)  86 (78.9) | 40 (23.3)  23 (21.1) | 1  0.91 (0.58–1.43) | 0.673 |
| Night shift  No  Yes | 109 (79.6)  104 (75.9) | 28 (20.4)  33 (24.1) | 1  1.18 (0.76-1.84) | 0.468 |
| Time working at institution (years)  ≤ 5  > 5 | 111 (78.7)  101 (75.4) | 30 (21.3)  33 (24.6) | 1  1.16 (0.75-1.79) | 0.509 |
| Has a non-care activities in the institution  No  Yes | 179 (76.2)  31 (88.6) | 56 (23.8)  4 (11.4) | 1  0.48 (0.18-1.24) | 0.100 |
| Works out of Hospital  No  Yes | 141 (78.3)  76 (76.8) | 39 (21.7)  24 (23.2) | 1  1.07 (0.68-1.69) | 0.763 |
| Absenteeism  (last month)  No  Yes | 172 (78.9)  45 (72.6) | 46 (21.1)  17 (27.4) | 1  1.30 (0.80-2.10) | 0.293 |
| Tobacco use  No  Yes | 197 (77.2)  20 (83.3) | 58 (22.8)  4 (16.7) | 1  0.73 (0.29-1.84) | 0.493 |
| Alcohol use  < 1 drink/week  ≥ 1drink/week | 170 (79.8)  47 (70.1) | 43 (20.2)  20 (29.9) | 1  1.48 (0.94-2.33) | 0.099 |
| Leisure  < 5 hours/week  ≥ 5 hours/week | 128 (74.8)  86 (81.1) | 43 (25.2)  20 (18.9) | 1  0.75 (0.47-1.20) | 0.226 |
| Regular physical activity  No  Yes | 120 (77.4)  89 (77.4) | 35 (22.6)  26 (22.6) | 1  1.00 (0.64-1.56) | 0.996 |
| Regular hobbies  No  Yes | 93 (74.4)  85 (79.4) | 32 (25.6)  22 (20.6) | 1  0.80 (0.50-1.29) | 0.365 |
| Family support  No  Yes | 84 (73.0)  126 (80.2) | 31 (27.0)  31 (19.8) | 1  0.73 (0.47-1.13) | 0.161 |
| Psychotherapy  No  Yes | 205 (77.9)  12 (70.6) | 58 (22.1)  5 (29.4) | 1  1.33 (0.62-2.88) | 0.481 |
| Sexual activity  < 1 time/week  ≥ 1 time/week | 70 (79.5)  143 (76.5) | 18 (20.5)  44 (23.5) | 1  1.15 (0.71-1.87) | 0.569 |
| Moral distress^4^  No  Yes | 95 (85.6)  74 (71.1) | 16 (14.4)  30 (28.9) | 1  2.00 (1.16-3.44) | 0.010 |

^#^ Three professionals did not complete The Maslach Burnout Inventory.

¹ Prevalence ratio.

² 95% Confidence Interval.

³ Intensive Care Unit.

^4^ Information available for 215 respondents.

* Chi-square test.
